# The Impact of Lighting Conditions on Users' Alertness and Working Memory in Confined Spaces

**DOI:** 10.1002/pchj.70022

**Published:** 2025-06-10

**Authors:** Zaoyi Sun, Shenshen Xie, Shang Hu, Changhua Jiang, Shaowen Ding, Litao Wu, Weidan Xu, Hongting Li

**Affiliations:** ^1^ Zhejiang University of Technology Hangzhou China; ^2^ China Astronaut Research and Training Center Beijing China; ^3^ School of Business Administration(MBA) Zhejiang Gongshang University Hangzhou China

**Keywords:** alertness, confined space, lighting, working memory

## Abstract

Confined spaces, characterized by limited natural ventilation, the absence of windows, and restricted access to natural light, present distinct challenges. While most studies focus on lighting's effect on sleep, confined spaces are now more often used for short‐term work‐rest cycles, especially in office settings. This study explores how lighting conditions affect alertness, cognitive performance, and physiological metrics such as heart rate variability (HRV) during work‐rest cycles in confined spaces. Participants performed 2‐back tasks, psychomotor vigilance tasks (PVT), and completed subjective scales under six lighting conditions, combining two levels of illuminance (300, 500 lx) and three color temperatures (2800, 5000, 6500 K). Results show higher subjective alertness during work with 300 lx and 5000 K. However, lighting conditions did not significantly affect subjective alertness during rest. Objective alertness was better at 300 lx, with 2800 outperforming 5000 K. Working memory accuracy was higher at 5000 compared with 6500 K, and reaction times were faster under 300 lx. Physiological data remained consistent across lighting conditions. These findings can inform future lighting design and management in confined spaces to improve comfort and efficiency.

## 1 Introduction

“Confined space,” as officially defined by the Chinese standard GBZ/T 205—2007, “Occupational Hazard Protection Specification for Confined Space Operations,” refers to windowless spaces with restricted access, limited natural ventilation, and the absence of natural light, solely relying on artificial lighting for illumination (Ko et al. 2022; Wang et al. 2022). Such spaces are prevalent in military contexts such as submarines, manned spacecraft, and lifeboats, where individuals operate in fully enclosed settings (Gou et al. 2025). Due to their unique characteristics, confined spaces pose inherent risks, especially during prolonged work when access to natural sunlight is limited. Hence, artificial lighting becomes essential not only for maintaining visibility but also for supporting circadian rhythm regulation (Dong et al. 2021). However, beyond such specialized contexts, confined spaces are increasingly utilized in industrial contexts for daily work and short‐term rest (Gou et al. 2023). This controlled setting simulates an enclosed space designed for typical daily work activities and short‐term rest, providing a platform to investigate the impact of lighting conditions on individuals' cognitive performance and emotional states.

Extensive research has demonstrated that lighting conditions influence not only visual performance but also physiological states, psychological well‐being, and cognitive abilities (Gerhardsson and Laike 2021). A recent review on enclosed cabins emphasizes the importance of evaluating lighting environments from both visual and nonvisual perspectives (Gou et al. 2023). Prolonged exposure to confined spaces without daylight has been linked to circadian rhythm desynchrony (Caddick et al. 2017) and sleep disorders (Dunster et al. 2023). Evidence suggests that targeted lighting interventions can help stabilize circadian rhythms and facilitate adaptation to shift work in such environments (Wang et al. 2022). In terms of psychological and behavioral outcomes, insufficient exposure to high‐intensity light during daytime hours in confined spaces has been associated with cognitive decline and heightened negative emotions (Wang et al. 2024). To address these issues, studies have shown that well‐designed lighting environments can enhance visual performance (Wang et al. 2023) and boost subjective alertness (Xiao et al. 2024), thereby mitigating disruptions to biorhythms in enclosed settings.

The effects of light on cognition are influenced by multiple factors, with illuminance level and correlated color temperature (CCT) being two of the most frequently analyzed features of interior lighting. Extensive research conducted in non‐confined spaces has highlighted the impacts of CCT and illuminance on cognitive functions such as alertness, inhibitory control, and working memory (Kang et al. 2019; Sun and Lian 2016). In confined spaces, however, the effects of light interventions on alertness remain inconsistent. One meta‐analysis reported that bright light exposure significantly enhanced subjective alertness compared with dim light conditions in enclosed environments (Li, Fang, Guo et al. 2024). Conversely, another recent meta‐analysis found no significant differences in either subjective or objective alertness between high‐intensity and low‐intensity light exposure (Mu et al. 2022). Research on the influence of illuminance and CCT on working memory performance in confined spaces is limited. Li, Fang, Qiu, and Wang (2024) found that under dynamic lighting conditions with increased illuminance and CCT (from 4000 K, melanopic equivalent daylight illuminance (EDI) = 224 lx, to 12,000 K, EDI = 420 lx), participants demonstrated shorter reaction times in n‐back tasks compared to baseline conditions (4000 K, EDI = 224 lx), while task accuracy remained unaffected. Moreover, Ru et al. (2021) reported that the effects of light on working memory were moderated by task type and difficulty.

Simulated space mission studies primarily focus on the effects of dynamic lighting schemes, adjusted across different time periods, on circadian rhythms and task performance (e.g., Grant et al. 2024; Rahman et al. 2022). These studies largely emphasize the influence of lighting on sleep‐related physiological rhythms, which is critical for understanding long‐term physiological adaptations in confined environments. As confined spaces increasingly serve diverse purposes, including use as enclosed office environments in certain scenarios, individuals may engage with these spaces during daytime for short‐term work‐rest cycles rather than extended stays. This study explores how different lighting conditions affect subjective and objective alertness, cognitive task performance, and physiological metrics such as heart rate variability (HRV) during work‐rest cycles in enclosed settings.

### 1.1 The Impact of Different Lighting Conditions on Users' Alertness

Alertness is defined as the state of being awake, aware, attentive, and prepared to respond (Vandebos 2007). Subjective alertness refers to an individual's self‐perceived level of alertness, typically assessed using self‐reported scales like the Karolinska Sleepiness Scale (KSS). In contrast, objective alertness is measured through cognitive performance tasks, such as the psychomotor vigilance task (PVT), which provide quantifiable data on reaction time, attention, and other cognitive functions (Cajochen 2007). Previous research has indicated a correlation between alertness and heart rate. For instance, Askaripoor et al. (2018) found that higher heart rate variability correlates with heightened levels of alertness, suggesting that heart rate can mirror cognitive and emotional states. In confined extreme environments, a recent meta‐analysis found that daytime electric lighting intervention was found to affect subjective alertness, PVT performance, and EEG activity related to conscious alertness levels (Li, Fang, Qiu, and Wang 2024). Illuminance, defined as the light flux density received per unit area, depends on factors such as brightness, distance of the light source, and position and direction of the illuminated surface (Ashdown 1993). Research on the impact of illuminance on alertness and other higher cognitive processes presented mixed findings, indicating positive, negative, and neutral effects (Siraji et al. 2022). While some studies reported that increased illuminance levels enhance subjective and objective alertness, vitality, sustained attention in tasks, and heart rate variability (Smolders et al. 2012), other studies found no significant difference in PVT task performance between high illuminance (1000 lx) and low illuminance (100 lx) lighting (Ru et al. 2019). A recent meta‐analysis found that the effect of exposure to high‐intensity light on both subjective and objective alertness was not significantly different from that of exposure to low‐intensity light (Mu et al. 2022).

Correlated color temperature (CCT) describes the color characteristic of light emitted by a source, based on its comparison to a theoretical blackbody radiator, and is measured in Kelvins (K). Light sources with higher CCT values (≥ 4000 K) tend to emit a bluish‐white light, often referred to as cooler tones, while those with lower CCT values (≤ 3000 K) produce a reddish‐white light, typically associated with warmer tones (Boray et al. 1989). One reason for the influence of CCT on subjective alertness is that CCT may also vary the color rendition on task surfaces. From chromatic stimuli, this can be significant, as the light source may not render colors accurately, reducing visual performance for color‐based tasks. For example, low and warmer color temperatures may not effectively render red stimuli, thereby reducing subjective alertness (Liang et al. 2021). Research has shown that lighting with higher CCT can enhance both subjective and objective alertness. For example, Mills et al. (2007) found that exposure to light at 17000 K significantly enhanced alertness and performance metrics compared to lower CCT levels of 4000 K or 2900 K. Similarly, Li, Fang, Qiu, and Wang (2024) reported that for railway dispatchers working in a confined control center, higher CCT lighting (12,000 K) supported subjective alertness and positive emotions. Additionally, EEG data from this study revealed that higher CCT lighting partially mitigated the decline in alertness and cognitive performance caused by mental fatigue.

Conversely, other studies have reported no significant impact of CCT on alertness. Smolders and De Kort (2017) discovered no benefits of higher CCT levels on attention, orientation, or executive control in their research. Additionally, individual preferences for CCT can vary depending on the purpose of the environment and the specific activities performed within it (Park et al. 2010).

The interaction between illuminance and CCT on alertness has also yielded mixed findings in the literature. For instance, Ru et al. (2019) investigated the diurnal effects of illuminance levels (100 vs. 1000 lx at eye level) and correlated color temperature (3000 vs. 6500 K) in a simulated office environment on subjective alertness. Their study found no significant main effects or interaction effects between illuminance and CCT. Conversely, Souman et al. (2018), in a review of 68 studies, demonstrated that higher intensities of polychromatic white light were frequently associated with increased subjective alertness. Supporting this, Chen et al. (2022) found that warm colors combined with bright lighting (3000 K, 590 lx) significantly enhanced both subjective and objective alertness. In summary, while lighting with higher CCT has the potential to enhance alertness, the relationship is intricate and affected by multiple variables. Consequently, our study seeks to delve deeper into these complexities to formulate more effective lighting strategies aimed at improving alertness in confined spaces.

### 1.2 The Impact of Different Lighting Conditions on Users' Working Memory Performance

Working memory involves a series of cognitive processes that enable the active retention of information to support decision‐making (Owen et al. 2005). An uncomfortable work environment can contribute to cognitive fatigue and diminished performance, which in turn increases mental workload, particularly during tasks requiring working memory. Previous research has yielded mixed findings regarding the impact of daytime exposure to high illuminance on working memory, with some studies reporting positive, null, or even negative effects. For instance, Kretschmer et al. (2011) examined the influence of lighting in a confined workspace (5.2 m × 7.4 m × 3.8 m) using the N‐back task to measure changes in working memory among older adults under conditions of intense light (3000 lx) and standard working light (300 lx). The findings revealed no significant difference in response times between the lighting conditions, but task accuracy was notably improved under 3000 lx. Conversely, Smolders and Kort (2014) found that higher illuminance levels (1000 lx) were associated with a reduced percentage of correct responses in the 2‐back task compared to lower illuminance (200 lx). These results suggest that illuminance manipulation during the day may offer selective advantages for working memory performance. Moreover, it has been suggested that the impact of illuminance on cognitive function may be moderated by both types of cognitive task and task difficulty (Konstantzos et al. 2020; Ru et al. 2021). In another study, Huiberts et al. (2015) conducted a laboratory experiment utilizing N‐back tasks of varying difficulty (2‐back and 3‐back) to assess participants' working memory under two lighting conditions: 200 and 1000 lx. Despite their efforts, no significant effects were observed. Much of the research on how lighting intensity affects cognitive performance has focused on non‐confined or workplace environments. Therefore, further empirical investigation is necessary to enhance our understanding of this relationship.

Studies dealing with the CTT of light and working memory show more mixed results. Some studies found that the blue‐enriched light environment decreases sleepiness and promotes alertness, thereby improving working memory performance (e.g., Chellappa, Steiner et al. 2011). For example, Ye et al. (2018) found that participants performed better in the 2‐back task under lighting of a higher CTT range. Zhu et al. (2019) manipulated both CCT and illuminance levels to examine their combined effects on task performance under natural daytime conditions. Conversely, other studies have found that lower CCTs do not significantly impair working memory but create a more relaxed and comfortable environment (Hygge and Knez 2001). The blue‐filtered light environment may lead to more melatonin and slower reaction speed (Vethe et al. 2021). One of the reasons for the diverging results in these studies is that some employed blue‐enriched light, which is full‐spectrum and appears white, whereas others manipulated high CCT values. Consequently, a more nuanced exploration is needed, considering confined space scenarios and varying CCT levels.

The interaction between illuminance and CCT on cognitive function has also been explored in studies focusing on nocturnal light environments. Nie et al. (2024) reported that a nocturnal light environment with lower CCT and higher illuminance effectively enhanced cognitive performance in the evening and improved subsequent sleep quality. However, a recent review by Liu et al. (2024) highlighted that high illuminance and high CCT are not universally beneficial for all cognitive tasks. Their findings suggest that certain cognitive performance tests have specific thresholds for optimal lighting conditions, which vary depending on the task. Furthermore, while different cognitive tasks require tailored lighting environments, selecting a neutral CCT (3300–5300 K) appears to be a generally effective approach for improving cognitive performance. These findings underscore the importance of exploring lighting design strategies in confined spaces, particularly in relation to tasks involving working memory.

### 1.3 The Impact of Different Lighting Conditions on Users' HRV

Heart rate variability (HRV), defined as the fluctuation in intervals between adjacent heartbeats (RR intervals), reflects the autonomic nervous system's (ANS) ability to regulate cardiovascular function. Light signals, transmitted via the retina's intrinsically photosensitive retinal ganglion cells (ipRGCs) to the hypothalamic suprachiasmatic nucleus (SCN) (Hankins et al. 2008), influence parasympathetic output to the heart through pre‐autonomic neurons within the hypothalamus. This mechanism facilitates the 24‐h sympathetic–parasympathetic balance of cardiac autonomic inputs (Kalsbeek et al. 2011). Consequently, the specific characteristics of lighting—such as illuminance and correlated color temperature (CCT)—have a pronounced regulatory effect on HRV.

Research into the effects of illuminance on HRV has yielded mixed findings (Chellappa et al. 2017). On one hand, higher illuminance levels (e.g., 1000 lx of bright polychromatic white light) have been shown to increase the low‐frequency to high‐frequency (LF/HF) HR power ratio compared to baseline or lower illuminance conditions (e.g., 200 lx), indicating a relative increase in cardiac sympathetic activity (Smolders et al. 2012). This suggests that higher illuminance during the daytime can enhance alertness and attention levels. Conversely, lower illuminance levels have been associated with increased parasympathetic activity, promoting relaxation and recovery. For example, Luo et al. (2022) observed that under lower illuminance conditions (e.g., 1200 or 200 lx) of blue light environments (6500 K), time‐domain HRV indices, such as RMSSD, showed significant improvement, whereas frequency‐domain indices remained unchanged. Moreover, a recent systematic review indicated that high melanopic equivalent daylight illuminance (EDI) was not more strongly associated with an increased heart rate than other α‐opic EDIs, suggesting that different spectral compositions of light may not differentially influence autonomic regulation (Leveille et al. 2025).

Regarding the influence of CCT on HRV, Araujo et al. (2020) demonstrated that red light exposure facilitated HRV relaxation, whereas white light elicited an active heart rate response. Other studies have found that higher CCT compared to lower CCT positively affects alertness and cognitive performance (Burattini et al. 2019; Chellappa, Gordijn et al. 2011). However, inconsistent findings exist, with some studies reporting no significant effects of CCT on alertness or cognitive performance (Lasauskaite et al. Lasauskaite and Cajochen 2018). These discrepancies may stem from variations in exposure duration, time of day, experimental methods, and spectral composition (Zhang et al. 2024).

Studies examining the combined effects of illuminance and CCT on HRV remain limited. Seo and Kim (2015) conducted an experiment in an enclosed space with three illuminance levels (50, 150, 300 lx) and three CCT levels (2000, 3800, 5600 K). They observed that at higher illuminance (300 lx), LF values were lowest under CCTs of 2000 and 3800 K, while HF values were highest under a CCT of 5600 K. Moreover, HF showed minimal activation under 2000 and 3800 K at 300 lx, but increased significantly as illuminance rose under 5600 K. These findings suggest that the combination of illuminance and CCT significantly impacts HRV. High illuminance and high CCT conditions may enhance sympathetic nervous activity, whereas low illuminance and low CCT conditions may promote parasympathetic activity. However, this study did not include task‐based conditions, limiting its ability to differentiate the effects of lighting on HRV during task engagement versus rest.

In summary, while previous studies have provided valuable insights into the effects of lighting on sleep‐related physiological rhythms in confined spaces, their focus has primarily been on long‐term adaptations. This study shifts the perspective to explore how lighting conditions influence short‐term work‐rest cycles in these contexts. Notably, prior research has examined the independent effects of illuminance and correlated color temperature (CCT) on subjective perception and physiological responses. However, the interaction between these factors, where one may dominate under specific conditions, has often been overlooked. To address this gap, the present study investigates the combined effects of illuminance and CCT on subjective perception, physiological health, and cognitive performance in confined spaces. Using a factorial design, we independently manipulated illuminance and CCT and assessed their impact through an objective alertness task (psychomotor vigilance task, PVT), a subjective alertness scale, and a working memory task (n‐back). By examining the effects of electric lighting in both work and relaxation settings, this study aims to optimize lighting conditions in confined environments to enhance work efficiency and the subjective well‐being of individuals operating in such spaces.

## 2 Method

### 2.1 Participants

Using G*power3.1 software to calculate the sample size required for the study, effect size *f* = 0.25, *α* = 0.05, 1—*β* = 0.95, it was calculated that a single experiment required 16 participants. The study recruited 30 participants, including 16 males, with ages ranging from 21 to 36 years (*M* = 28.2, SD = 3.97). All participants were affiliated with the China Astronaut Research and Training Center. Eligibility criteria included normal or corrected‐to‐normal vision, absence of color blindness or color deficiency, right‐handedness, and no prior experience with similar experiments. Ethical approval was obtained from Zhejiang University of Technology's Human Research Ethics Committee. All participants provided written informed consent and received a compensation of 200 RMB upon completion.

### 2.2 Design

**FIGURE 1:**
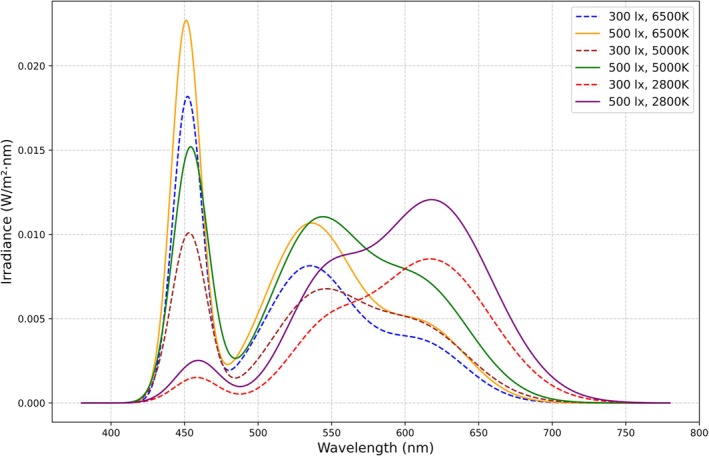
The spectral power distribution.

The study employed a 2 (Illuminance: 300, 500 lx) × 3 (Color Temperature: 2800, 5000, 6500 K) within‐subjects design. The chosen combinations are supported by the recommendations of GB 50034–2013 and the practical applications of ISS lighting systems. The Chinese National Standard GB 50034–2013 provides detailed guidance on indoor lighting design, including recommended ranges for illuminance levels and CCT to meet different functional and visual comfort needs: For general office environments, the recommended illuminance levels range from 300 to 500 lx, representing medium to high lighting levels that support tasks such as reading, writing, and computer work. The ISS lighting system emphasizes the impact of lighting on circadian rhythms (Brainard et al. 2016), cognitive performance, and mental health. It includes three key lighting modes: General lighting (4500 K, 210 cd): Provides a balanced light environment for daily activities. Alertness lighting (6500 K, 420 cd): Incorporates blue‐rich white light to enhance alertness and focus. Sleep preparation lighting (2700 K, 90 cd): Reduces blue light to promote relaxation and sleep. The selected high CCT (6500 K) and low CCT (2800 K) conditions in the experiment correspond to ISS lighting modes for alertness enhancement and short relax, respectively. The spectrally weighted α‐opic EDIs of light and the relative spectral power distributions are shown in Table 1 and Figure 1, respectively. The lighting parameters were reported in the ENLIGHT checklist, as shown in Table 2 (Spitschan et al. 2023, Table 2).

**TABLE 1:** Spectrally weighted *α*‐opic equivalent daylight illuminance (EDI) at eye level for each light condition.

	2800 K		5000 K		6500 K	
	300 lx	500 lx	300 lx	500 lx	300 lx	500 lx
Melanopic	150	249	205	341	261	436
S‐cone	121	201	210	349	268	448
M‐cone	225	375	240	397	292	487
L‐cone	278	467	265	443	272	452
Rhodopic	165	275	222	370	270	450

*Note:* The *α*‐opic (melanopic, rhodopic, S‐cone, M‐cone and L‐cone) EDI in lx were determined using the calculation toolbox developed by CIE.

**TABLE 2:** ENLIGHT checklist.

Item	Description of the study
A. Study characteristics	
A.1. Protocol‐level characteristics	
Description of experimental setting	The experimental space measured approximately 2.5 m × 1.8 m × 1.6 m, consisting of a table and a chair, with participants facing one side wall (1.8 m × 1.6 m), all four walls are white. A 14‐in. laptop was centrally positioned on the table. The ambient temperature was maintained at 25°C, and humidity ranged from 40% to 60%.
Pre‐laboratory sleep–wake/rest‐activity behavior	Ensure a regular sleep schedule.
Pre‐laboratory light exposure	Without any light source.
Immediate prior light exposure (in laboratory)	Yes
A.2. Measurement‐level characteristics	
Measurement plane	Vertical
Measurement viewpoint and location	Spectrometer placed vertically in the middle of the eyes and along the viewing direction of the participants.
Type, make and manufacturer of the measurement instrument	Type: Illuminance meter Make: EVERFINE. Manufacturer: Everfine Photometric Co. Ltd. (based in China).
Calibration status of the instrument	The instrument has been calibrated.
A.3. Participant‐level characteristics	
Ocular health and functioning	The participants have normal ocular health and functioning.
Pupil size and/or dilation	Pupil size is within the normal range and responds appropriately to light.
Relative time	The participants perceives time in a typical manner.
B. Light characteristics	
B.1. Light source type(s)	Room illumination (overhead or other), Polychromatic
Type, make and manufacturer of the light source	Type: LED lamp Make: MJDP003 Manufacturer: Qingdao Yilianke Information Technology Co. Ltd. (Xiaomi Ecological Chain Enterprise) Address: B4, 10th Floor, Building B, Qingdao International Innovation Park, No. 1, Weiyi Road, Keyuan, Laoshan District, Qingdao
Use of wearable filtering apparatus (e.g., blue‐blocking glasses)	None of the participants reported using wearable filtering apparatus like blue‐blocking glasses.
B.2. Light level characteristics	
Illuminance (lux) and/or luminance (cd/m2)	The luminous flux of a single light source ranges from 25 to 500 lm. With 12 light sources in use, the illuminance range is 33.33–1333.33 lx
B.3. Color characteristics	
Color appearance quantities	2700–6500 K
B.4. Temporal and spatial characteristics	
Location of stimulus and viewing distance	The stimulus was displayed at the center of the screen, with the participant positioned at a viewing distance of approximately 50 cm.
Relative or absolute size of the stimulus	The stimulus was displayed at the center of the screen.

The dependent variables included subjective ratings on a scale, reaction time in the psychomotor vigilance task (PVT) (Basner et al. 2011), reaction time, accuracy in the N‐back task, and heart rate variability. Control variables included ambient temperature and humidity.

### 2.3 Equipment and Materials

#### 2.3.1 Equipment

**FIGURE 2:**
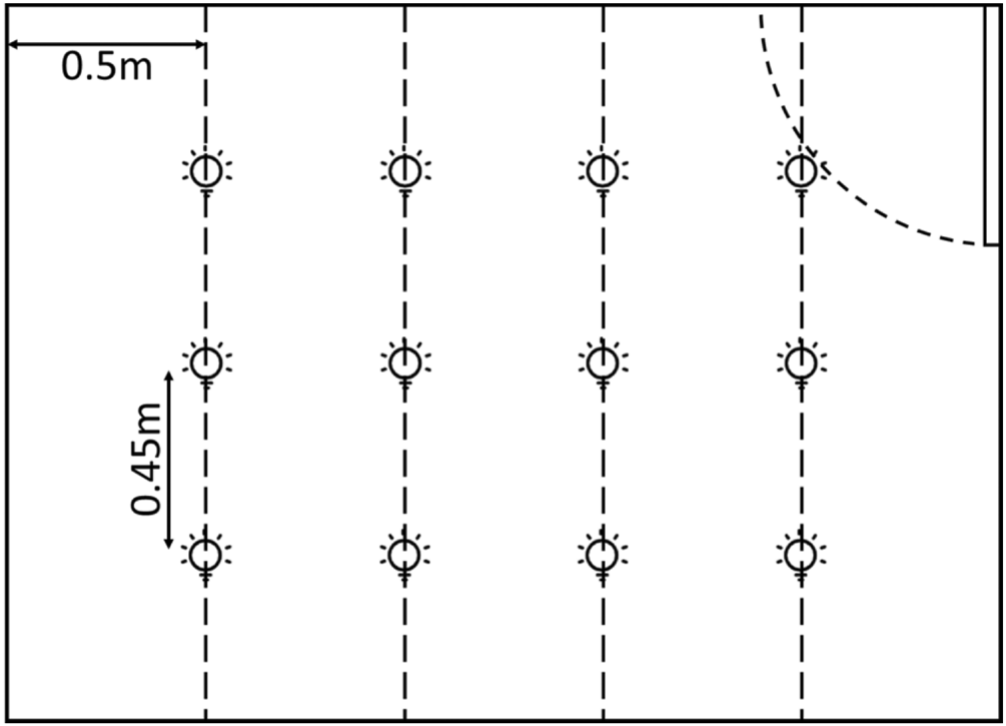
Arrangement of lights.

The study was conducted in a laboratory using a Windows 10 computer system to present experimental tasks and record participant inputs. 12 LED lights (Xiaomi LED Smart Bulbs Bluetooth MESH version) with adjustable CTTs ranging from 2700 K to 6500 K controlled illuminance levels were used. As shown in Figure 2 the lights were arranged in a 4 × 3 grid pattern, with a horizontal spacing of 0.5 m and a vertical spacing of 0.45 m between adjacent lights. This arrangement provided consistent illumination within the laboratory space, minimizing shadows or uneven lighting. Illuminance was measured using a spectrometer (EVERFINE, SPIC‐200) positioned vertically at the participants' eye level and aligned with their viewing direction to ensure accurate representation of the lighting conditions as perceived by the participants.

#### 2.3.2 Environmental Settings

**FIGURE 3:**
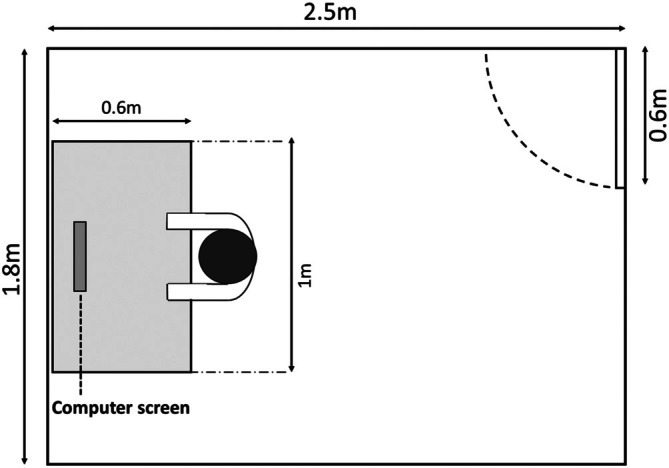
Laboratory layout.

**FIGURE 4:**
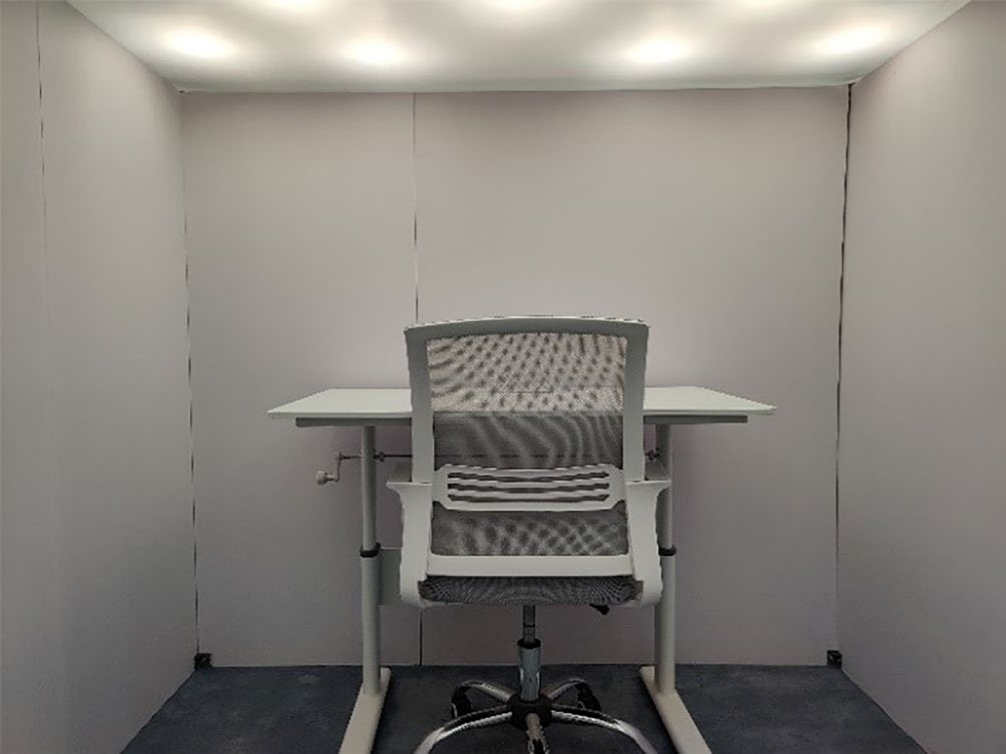
Experimental scene.

The experimental space measured approximately 2.5 m × 1.8 m × 1.6 m, consisting of a table and a chair, with participants facing one side wall (1.8 m × 1.6 m); all four walls are white, as illustrated in Figure 3. A 14‐in. computer with a resolution of 1920 × 1080 was used to present experimental materials and placed at the center of the booth bottom. The white luminance of the display was approximately 169 cd/m^2^. The ambient temperature was maintained at 25°C, and humidity ranged from 40% to 60%. The experimental environment is depicted in Figure 4.

### 2.4 Experimental Tasks

#### 2.4.1 Psychomotor Vigilance Task (PVT)

The standard PVT task typically lasts 10 min with 80 trials. In this study, we used a brief version of the PVT, which lasts 5 min and consists of 40 trials. Specifically, participants were required to respond to a red dot stimulus presented at a variable interval (2000–10,000 msec) by pressing the “space” button. Several studies have demonstrated the feasibility of using shorter versions of the PVT (Loh et al. 2004), which can enhance the practicality and user acceptance of the task compared to the standard 10‐min version. The alertness index was determined based on the average reaction time.

#### 2.4.2 2‐Back Task

**FIGURE 5:**
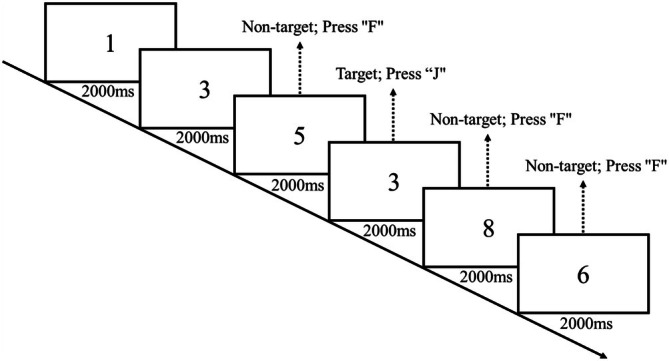
2‐back task diagram.

The 2‐back task, implemented using E‐Prime software, was employed to assess working memory performance, with stimuli comprising the digits 0–9. During the task (Figure 5), participants were instructed to determine whether each stimulus in a sequence matched the one presented two positions earlier (Buschkuehl et al. 2014). Participants were instructed to respond as quickly and accurately as possible. The “F” and “J” keys were used to indicate “no” and “yes” responses, respectively. Each experimental condition consisted of 114 trials, and participants were required to respond within the duration of the stimulus presentation.

### 2.5 Measurements

#### 2.5.1 Subjective Alertness Assessment Scale

The Karolinska Sleepiness Scale (KSS), developed by Åkerstedt and Gillberg (1990), is employed in this study to assess participants' alertness levels and subjective sleepiness. The KSS classifies an individual's alertness into nine grades (1 = *extremely alert*, 9 = *extremely sleepy*), with higher scores indicating lower alertness levels. For this study, the KSS scale has been translated into Chinese to facilitate participants in self‐assessing their levels of sleepiness.

### 2.6 Procedure

The experiment is structured into two distinct stages: a practice session and a formal experiment. In the initial phase, participants undergo training in both the PVT and 2‐back tasks to familiarize themselves with the experimental protocol.

**FIGURE 6:**
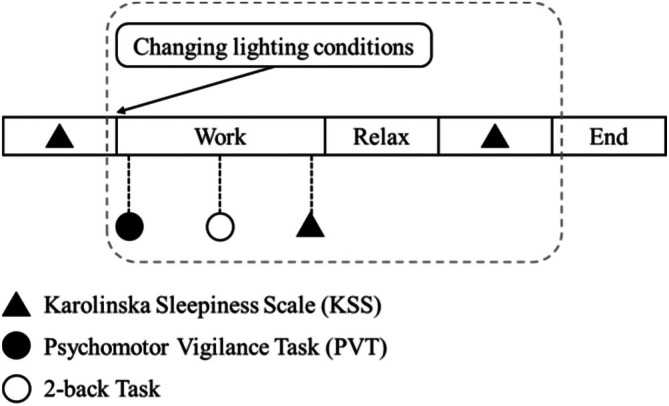
The sequence of tasks for each condition in the formal experiment.

In the formal experiment, participants performed the tasks in a fixed sequence: the PVT task was administered first, followed by the 2‐back task under each experimental condition. After the completion of each task, a questionnaire appears on the screen, mandating the participants to provide their responses. The six experimental conditions are presented in a Latin square order to counterbalance sequence effects. A Latin‐square design was used to assign six experimental conditions to participants, with the detailed order provided in Table S1. Figure 6 shows the sequence of tasks for each condition; six sequences of lighting conditions were repeatedly assigned across the 30 participants. Subsequent to task completion, participants are instructed to maintain a quiet, restful state for 5 min within the same lighting environment and complete the KSS questionnaire. Participants' HRV was measured throughout the task. Each experimental condition requires approximately 20 min, with a 2‐min interval between experimental conditions, contributing to a total experiment duration of approximately 1.5 h. In this study, the experiments are conducted from 9:00 AM to 6:00 PM.

### 2.7 Data Analysis

HRV data were collected and analyzed using the PhysioLab software. The software computes the average heart rate (HR) and displays it on the feature panel. HRV analysis is conducted by assessing the regulation of heart activity through the variation of R peaks over time. Given the essential role of RR interval editing in HRV analysis (Albinet et al. 2010), PhysioLab supports this process in two main ways: (a) it allows users to filter HR data within a specific range (default 30 to 150 BPM), and (b) it detects and removes outliers, defined as HR values deviating from the preceding value by more than a set multiple of the standard deviation (default *n* = 2, as suggested by Muñoz et al. 2016). RR interval correction is performed prior to the calculation of both time‐domain and frequency‐domain HRV parameters. Detailed formulas for these calculations are provided in Table S2.

The main effects and interaction effects of illuminance and CCT were analyzed using SPSS 26.0 software. Post hoc comparisons were performed using the Bonferroni correction to adjust for multiple comparisons and ensure the robustness of the findings. During the experimental tasks, which included the PTV task and the 2‐back task, each trial had a fixed duration. Trials in which participants failed to respond within the fixed time limit were automatically terminated and excluded from the analysis. Additionally, in the analysis of the 2‐back task, responses faster than 200 ms were excluded from the reaction time (RT) calculation, following the standard applied in Scharinger et al. (2015). This approach ensured the reliability of the data and minimized the impact of spurious responses on the results. To further explore the subtleties of the data, paired‐sample *t*‐tests were used specifically to assess HRV indicators under both work and rest conditions.

## 3 Result

### 3.1 Effects of Illuminance and Color Temperature on Subjective Alertness

**FIGURE 7:**
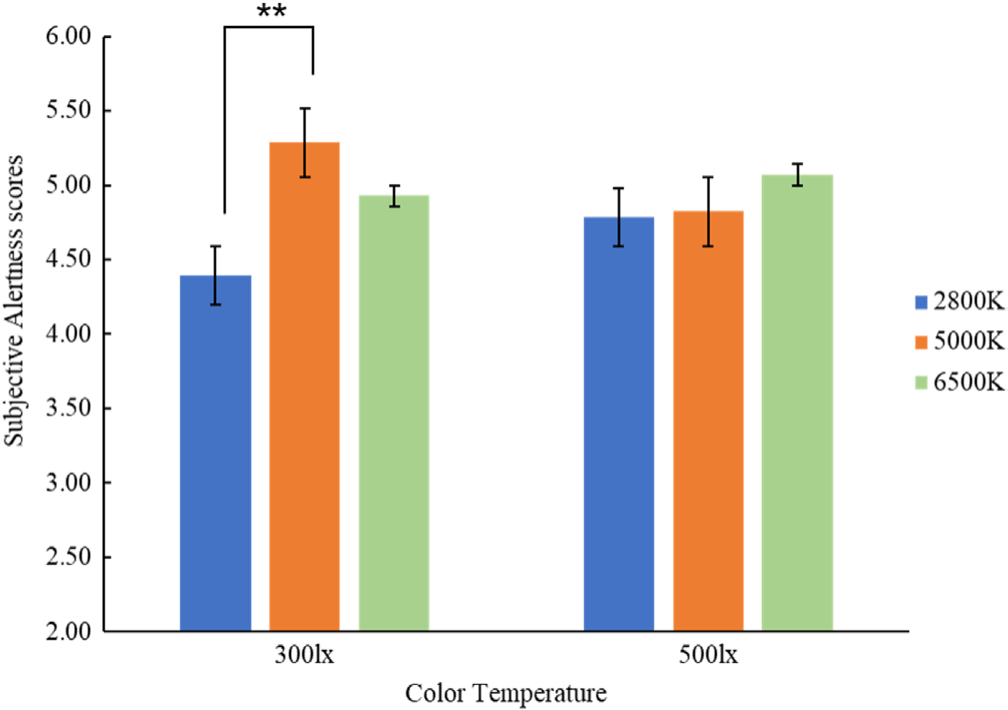
Subjective alertness levels across varying illuminance and color temperature.

After excluding the two groups with missing data, Table 3 presents the descriptive statistics for subjective alertness scores across various experimental conditions. Separate repeated measures analyses of variance were conducted for participants' subjective alertness in both work and relaxation states. In the working state, Mauchly's test indicated that sphericity is violated for CTT (*p <* 0.05), and therefore the *p* values of the RM‐ANOVA are corrected with the Greenhouse–Geisser correction. The main effect of illuminance was found to be nonsignificant [*F* (1, 27) = 0.01, *p* = 0.950]. Conversely, the main effect of CTT showed marginal significance [*F* (2, 54) = 2.48, *p* = 0.093, *η*_*p*_^2^ = 0.08]. Specifically, at 5000 K CTT (*M* = 5.05, SD = 0.19), the alertness level surpassed that at 2800 K (*M* = 4.59, SD = 0.19) and 6500 K (*M* = 5.00, SD = 0.25). The interaction between the two factors was also marginally significant [*F* (2, 54) = 2.85, *p* = 0.067, *η*_*p*_^2^ = 0.10]. Further examination through simple effect analysis revealed that at 300 lx illuminance, participants' alertness level was significantly lower at 2800 K compared with 5000 K (*p* = 0.012 < 0.05), 95% CI = [−1.62, −0.17]. However, no significant difference in alertness level was observed under different CTTs when the illuminance was 500 lx (Figure 7). In the relaxation state, neither the main effect of illuminance [*F* (1, 27) = 0, *p* = 1.000] nor the main effect of CTT [*F* (2, 54) = 2.05, *p* = 0.138] reached statistical significance. Additionally, no significant interaction effect between the two factors was found [*F* (2, 54) = 1.33, *p* = 0.272].

**TABLE 3:** Subjective alertness scores across varying illuminance and color temperature.

Illuminance	Color temperature		
	2800 K	5000 K	6500 K
300 lx	4.68 ± 1.63	5.32 ± 1.77	4.71 ± 1.78
500 lx	4.75 ± 1.35	4.96 ± 1.40	5.04 ± 1.63

*Note:* Subjective alertness scores are measured in milliseconds (*M* ± SD).

### 3.2 Effects of Illuminance and Color Temperature on N‐Back Task Accuracy and Reaction Time

A total of 23 valid datasets were collected, revealing an overall average accuracy of 92.69% with a standard deviation of 5.24%. Descriptive statistics were used to examine the average accuracy under varying illuminance and CTT (see Table 4). Mauchly's test indicated that sphericity is verified (*p* > 0.05), a repeated measures analysis of variance indicated that the main effect of illuminance lacked significance [*F* (1, 22) = 0.01, *p* = 0.935, *η*_*p*_^2^ = 0.00]. Conversely, the main effect of CTT showed marginal significance [*F* (2, 44) = 2.57, *p* = 0.088, *η*_*p*_^2^ = 0.11]. Notably, higher accuracy was observed at 5000 K (*M* = 93.32%, SD = 0.82%) than at 6500 K (*M* = 91.76%, SD = 1.10%) (*p* = 0.050). No significant interaction effect was observed between illuminance and CTT [*F* (2, 44) = 0.26, *p* = 0.776, *η*_*p*_^2^ = 0.01]. The mean reaction time was 718.41 ms with a standard deviation of 173.24 ms. Descriptive statistics were calculated for the average reaction time across varying illuminance and CTT levels (see Table 5). Mauchly's test indicated that sphericity is verified (*p* > 0.05); a repeated measures analysis of variance indicated a significant main effect of illuminance [*F* (1, 22) = 4.81, *p* = 0.039 < 0.05, *η*_*p*_^2^ = 0.18], signifying a notable increase in reaction time with heightened illuminance. Specifically, participants exhibited faster reaction times in the N‐back task at 300 lx (689.26 ± 152.99) than at 500 lx (747.55 ± 187.99). However, the main effect of CTT did not reach significance [*F* (2, 44) = 0.21, *p* = 0.811], and no significant interaction effect was observed [*F* (2, 44) = 0.71, *p* = 0.496].

**TABLE 4:** Accuracy of the N‐back task under different illuminance and color temperature conditions (*M* ± SD).

Illuminance	Color temperature		
	2800 K	5000 K	6500 K
300 lx	93.19% ± 0.89%	92.98% ± 0.96%	91.99% ± 1.43%
500 lx	92.90% ± 1.00%	93.78% ± 0.097%	91.64% ± 1.26%

*Note:* Accuracy of the N‐back Task is measured in milliseconds (*M* ± SD).

**TABLE 5:** Reaction time of the N‐back task under different illuminance and color temperature conditions (*M* ± SD).

Illuminance	Color temperature		
	2800 K	5000 K	6500 K
300 lx	676.42 ± 138.55	679.5504 ± 162.36	711.82 ± 161.19
500 lx	757.62 ± 196.78	749.0330 ± 204.65	736.00 ± 168.46

*Note:* Reaction time is measured in milliseconds (*M* ± SD).

### 3.3 The Average Reaction Time of the PVT Task

The mean average reaction time was 437.15 ms, with a standard deviation of 68.44 ms. Descriptive statistics were calculated for the average reaction time under various illuminance and CTTs (Table 6). Mauchly's test indicated that sphericity is violated for CTT (*p* < 0.05), and therefore the *p* values of the RM‐ANOVA are corrected with the Greenhouse–Geisser correction. Repeated measures analysis of variance revealed a significant main effect of illuminance [*F* (1, 29) = 11.54, *p* = 0.002 < 0.01, *η*_*p*_^2^ = 0.29]. As illuminance increased, there was a notable and significant rise in behavioral reaction time. Participants demonstrated faster reactions at 300 lx (*M* = 422.50, SD = 10.56) compared with the 500 lx condition (*M* = 451.80, SD = 11.87). The main effect of CTT was also found to be significant [*F* (2, 58) = 4.98, *p* = 0.010 < 0.05, *η*_*p*_^2^ = 0.15], indicating that reaction times were quicker at 2800 K (*M* = 426.83, SD = 10.25) than at 5000 K (*M* = 442.86, SD = 11.32) and 6500 K (*M* = 441.76, SD = 11.04). However, no significant interaction effect was observed between illuminance and CTT [*F* (2, 58) = 0.89, *p* = 0.415, *η*_*p*_^2^ = 0.03].

**TABLE 6:** Reaction time (*M* ± SD) across illuminance and color temperature conditions in the PVT task.

Illuminance (lx)	Color temperature (K)		
	2800	5000	6500
300	407.99 ± 53.43	441.66 ± 64.95	439.00 ± 80.43
500	479.40 ± 77.88	423.32 ± 61.15	431.54 ± 50.79

*Note:* Reaction time is measured in milliseconds (*M* ± SD).

### 3.4 The Variation of HRV Under Different Illuminance Conditions and Task States

Heart rate variability (HRV) serves as a noninvasive method to assess autonomic nervous system activity, using various measurable indicators. One key indicator is the standard deviation of normal‐to‐normal intervals (SDNN), which serves as a comprehensive measure of overall variability, reflecting the regulatory influence of both the sympathetic and parasympathetic nervous systems. Another metric, the average deviation of pulse intervals (rMSSD), is primarily indicative of parasympathetic regulation and serves as a sensitive short‐term variability marker and a reliable index of cardiac stability. It exhibits diminished values in states of anger, worry, and fear. NN50 signifies high‐frequency heart rate variability activity. High frequency (HF) primarily mirrors the influence of the parasympathetic nervous system, whereas low‐frequency (LF) captures the combined impact of both the sympathetic and parasympathetic nervous systems. The LF/HF ratio serves as an indicative measure of activity in the sympathetic and parasympathetic nervous systems, with a higher LF/HF ratio typically signaling increased sympathetic nervous system activity in comparison with the parasympathetic nervous system. In the context of this study, six indicators underwent repeated measures analysis, with participants maintaining a moderate level of alertness. Despite varying lighting intensities and CTTs, no significant differences were observed in these indicators.

Furthermore, the study scrutinized differences in HRV characteristics between work and rest conditions. The mean values of each HRV characteristic are outlined in Table 7. The results indicate that, except for NN50, HF, and LF/HF, all other HRV characteristics exhibited statistically significant differences (*p* < 0.05) between the work and rest conditions. Specifically, SDNN, rMSSD, and LF increased during the rest condition (see Table 8).

**TABLE 7:** ANOVA table for HRV indicators.

	Sources of Variation	SS	df	MS	*F*	*p*	*η*_*p*_^2^
SDNN	Illuminance	454.53	1	454.53	0.98	0.326	0.01
	CTT	903.05	2	451.52	1.74	0.117	0.02
	Illuminance × CTT	508.05	2	254.03	0.74	0.477	0.01
rMSSD	Illuminance	458.40	1	458.40	0.49	0.488	0.01
	CTT	495.71	2	247.85	0.53	0.589	0.01
	Illuminance × CTT	1388.79	2	694.40	1.04	0.356	0.01
pNN50	Illuminance	283.21	1	283.21	1.09	0.300	0.01
	CTT	332.23	2	166.11	0.78	0.460	0.01
	Illuminance × CTT	353.34	2	176.67	0.69	0.502	0.01
pLF	Illuminance	1847163.47	1	1847163.47	2.59	0.111	0.03
	CTT	3945775.58	2	1972887.79	1.21	0.300	0.01
	Illuminance × CTT	2863007.36	2	1431503.68	1.92	0.149	0.02
pHF	Illuminance	106.96	1	106.96	0.71	0.404	0.01
	CTT	306.63	2	153.32	1.29	0.278	0.02
	Illuminance × CTT	29.78	2	14.89	0.08	0.920	0.01
LF/HF	Illuminance	1.68	1	1.68	0.07	0.797	0.01
	CTT	95.01	2	47.50	1.94	0.147	0.02
	Illuminance × CTT	0.86	2	0.43	0.02	0.982	0.01

**TABLE 8:** Comparison of HRV indicators between working and resting states (*M* ± SD).

Indicators	Work	Relax	*t*	*p*	95% CI
SDNN	51.29 ± 11.57	69.04 ± 23.00	−5.30**	< 0.001	[−24.63, −10.87]
rMSSD	38.92 ± 14.71	53.00 ± 32.88	−2.64*	0.014	[−25.05, −3.11]
NN50	35.44 ± 27.13	37.02 ± 23.92	−0.91	0.374	[−7.21, 2.80]
LF	828.69 ± 534.26	1189.67 ± 885.89	−3.59**	0.001	[−567.75, −154.21]
HF	30.59 ± 6.96	29.95 ± 8.01	0.47	0.646	[−2.20, 3.49]
LF/HF	2.65 ± 2.06	2.78 ± 1.64	−0.30	0.764	[−0.98, 0.72]

*Note:* HRV Indicators is measured in milliseconds (*M* ± SD). Significance levels: *p < 0.05, **p < 0.01.

## 4 Discussion

This study investigated the effects of varying lighting conditions, defined by differences in light intensity and correlated color temperature (CCT), on subjective and objective alertness, working memory, and physiological indicators. The results revealed that subjective alertness during the working condition was influenced by the combined effects of light intensity and CCT, with the highest alertness observed under lighting conditions of 300 and 5000 K. However, subjective alertness during the resting condition remained unaffected by changes in lighting conditions. In terms of objective alertness, optimal performance occurred at a light intensity of 300 lx, while a CCT of 2800 K resulted in better performance compared to 5000 K. Working memory accuracy was significantly influenced by CCT, with higher accuracy rates recorded at 5000 K compared to 6500 K. Additionally, reaction times were influenced by light intensity, with faster responses recorded at 300 compared with 500 lx. However, physiological data showed no significant differences under varying lighting conditions. These findings suggest that both light intensity and CCT play important roles in modulating alertness and cognitive performance, while physiological responses appear less sensitive to these variations.

Previous research has demonstrated that both high‐intensity illumination and lighting with a high CCT can effectively suppress melatonin secretion, thereby enhancing individual alertness (Morita and Tokura 1996). Building on this foundation, the current study offers a more nuanced understanding of the boundary conditions that govern the effects of light intensity and CCT on individual alertness. Specifically, in a working state, participants reported the highest levels of alertness under lighting conditions with an intensity of 300 lx and a CCT of 5000 K. Notably, at an intensity of 500 lx, variations in CCT did not result in significant differences in subjective alertness. This suggests that, at relatively low light intensity, a CCT of 5000 K may be more effective in enhancing alertness. However, at higher light intensities, the impact of CCT on subjective alertness appears to be minimal. These results may be influenced by the role of the display and the confined room conditions. Although vertical illuminance at eye level was controlled in this experiment, research on visual ergonomics and workspace lighting suggests that variations in luminance contrast between task areas and peripheral surroundings significantly affect perceptions of a room (e.g., Durak et al. 2007). At a lower illuminance level (300 lx), softer and more diffused reflections from the white walls likely reduced the luminance contrast between the surroundings and the display. This softer visual environment may have mitigated visual fatigue, enhanced subjective comfort, and indirectly contributed to higher alertness. An alternative interpretation considers the circadian rhythm differences induced by lighting conditions. Smolders and De Kort (2017) examined the impact of one‐hour exposure at different times (morning vs. afternoon) under distinct CCT lighting environments (2700 vs. 6000 K) on subjective alertness. Their results revealed that while subjective vitality was higher in the morning under 6000 K lighting, there were no significant differences in wakefulness. In this study, participants were placed in an isolated space devoid of natural light exposure, and a CCT of 5000 K was chosen to approximate the natural light color during sunrise. The circadian rhythm changes induced by this specific CCT may contribute to the observed boundary effect. In contrast, when considering CCTs above 6000 K, existing literature suggests that blue light or light rich in blue tends to have a pronounced activating effect during darker evening or nighttime conditions (Chellappa, Gordijn et al. 2011). For example, at similar CCT levels but with lower illuminance (40 lx), exposure to blue‐enriched light late at night has been shown to enhance alertness. Moreover, research suggests that higher CCTs, particularly 6000 K, may evoke more unpleasant environmental feelings. This finding could explain the higher subjective alertness observed at 5000 K in our study (Fotios 2001). However, in examining the results during the rest phase, no significant effects were observed across different lighting conditions. This outcome may be attributed to the visual system's adaptation to a stable lighting environment over time, reducing sensitivity to changes in lighting conditions. Thus, it can be inferred that varying lighting environments may sufficiently meet the needs of individuals when resting in confined spaces with limited natural light exposure.

This study utilized the psychomotor vigilance task (PVT) as an objective measure of participants' alertness. Analysis of reaction time data revealed a notable distinction, with participants demonstrating quicker reactions under the 300 lx condition compared to the 500 lx condition. The PVT, designed to assess sustained attention, provides valuable insight into these findings. As light intensity increased to 500 lx, participants exhibited prolonged reaction times, suggesting a heightened fatigue effect under this condition. Contrary to the prevailing notion that intense light exposure invariably leads to acute activation of alertness and wakefulness (Cajochen et al. 2000; Rüger et al. 2005), these results underscore the nuanced interaction between light intensity and individuals' preceding mental states. Interestingly, the impact of strong light on alertness appears to intensify as the task progresses, consistent with previous studies (Smolders and de Kort 2014). In addition to light intensity, a significant association between CCT and reaction times was observed. Specifically, participants exhibited faster reaction times when the CCT was lower (2800 K). Existing literature on the effects of CCT on objective alertness suggests that, particularly during nighttime or low‐light conditions, higher CCT lighting may enhance both subjective and objective alertness compared to lower CCT lighting. This finding is supported by Chellappa, Steiner et al. (2011), who demonstrated significantly faster reaction times under 6500 K lighting compared to 2500 K and 3000 K white light under low‐light intensity conditions (40 lx). Chellappa, Steiner et al. (2011). However, the scenario changes during daylight hours, as studies consistently show that the effects of environmental lighting on alertness become less significant (Smolders and De Kort 2017). Divergent findings emerge, with some studies indicating that red light has a stronger alerting effect in the afternoon compared to blue light (Sahin and Figueiro 2013). The mechanisms through which lighting, including both intensity and CCT, affects alert remain complex. Light reaching the retina undergoes intricate neural processes with multifaceted effects on the brain. Moreover, the awakening effect of light may stem from a psychological response to neural processing of light on the retina. For instance, some studies propose that red light increases individuals' receptivity to external stimuli, fostering excitement and influencing emotional and behavioral responses (Hanford and Figueiro 2013; Danilenko et al. 2003). Future research is necessary to further explore the complex interplay between light characteristics and alertness mechanisms.

In this study, the N‐back paradigm was employed to assess working memory performance in individuals within confined spaces. Unlike the findings of Smolders et al. our results revealed no significant effect of light intensity on task accuracy. Smolders et al. observed higher accuracy in the 2‐back task under 200 lx conditions compared to 1000 lx conditions. Notably, the 200 lx condition in their study falls within the low‐light category (typically below 200 lx), whereas 1000 lx represents relatively high brightness. In contrast, our study utilized illuminances of 300 and 500 lx, both of which correspond to moderate and practical lighting conditions. It is possible that these illuminance levels were insufficient to produce statistically significant differences in task accuracy. Valdez et al. (2008) highlighted the complex interaction between environmental factors and task characteristics, such as task type and difficulty, in influencing working memory performance. Previous research also suggests that the effects of illuminance on working memory may depend on task difficulty. For example, Huiberts et al. (2015) found that higher illuminance levels did not significantly affect the accuracy of more challenging working memory tasks. In our study, the relative ease of the task for the participant group may explain the absence of significant differences in accuracy across varying lighting conditions. It is also plausible that participants prioritized response speed over accuracy as the experiment progressed, as indicated by the reaction time results. These findings align with those of Huiberts et al. (2015), who reported that participants performed better in working memory tasks under 200 compared with 100 lx after adjusting for office lighting CCT (4000 K). Their study suggests that both excessively low and high light intensities may impair working memory. Moreover, under low ambient illuminance in our study, the computer monitor used for task presentation—a self‐luminous display—likely interacted with the ambient light. The luminance and CCT of the display may have had a more prominent influence on the lighting conditions, potentially affecting cognitive performance. Future research should further investigate the interaction between low ambient illuminance and self‐luminous displays to better understand their impact on cognitive performance.

Additionally, this study found that CCT did not significantly affect the reaction time in the N‐back task. However, a notable improvement in task accuracy was observed when the CCT was set to 5000 K. Importantly, no significant differences in accuracy were found between the 2800 and 6500 K lighting conditions, which aligns with the findings of Ru et al. (2019). Their investigation, which examined the impact of high and low CCTs (6500 vs. 2800 K) on cognitive processing, reported no substantial effects of CCT levels. In our experiment, a deliberate choice was made to set the CCT level at 5000, a value between high and low, which resulted in improved performance under this specific condition. These results suggest that both excessively high and low CCTs in a confined, narrow space may hinder task performance. Some studies propose that prolonged exposure time is a critical factor for the beneficial effects of high‐CCT lighting to manifest. Notably, in our study, the overall exposure time to the lighting conditions was not sufficiently long, highlighting a potential limitation that warrants further investigation (Keis et al. 2014).

In addition to examining subjective reports and behavioral metrics, this study also explored the physiological indicators of participants. The results indicated no significant differences in various heart rate variability (HRV) measures among participants exposed to different lighting intensities and CCTs. This finding is consistent with Jiang et al. (2022), who investigated five CCT levels and found no significant variations in heart rate across different lighting conditions. Although some studies suggest that lighting at 6000 K can significantly elevate participants' heart rates during both day and night, our results differ, likely due to the brief duration of light exposure. Previous research indicates that short‐term lighting conditions may not substantially affect heart rate (Huiberts et al. 2016). Regarding lighting intensity, the two levels used in this study (300 and 500 lx) did not result in significant differences in HRV measures, suggesting that these lighting intensities elicit similar physiological arousal levels in participants. These physiological data imply that more pronounced differences in lighting conditions or longer exposure times may be necessary to observe notable effects on heart rate and to gain more robust insights into physiological responses.

This study also conducted a comparative analysis of physiological indicators in both working and resting states, revealing significant reductions in SDNN, rMSSD, and LF indices as participants transitioned from work to rest. Specifically, SDNN, a measure of the body's adaptive capacity, exhibited a notable decrease, indicating a reduction in heart rate variability. This decline suggests elevated stress levels during the work phase, reflecting a comparatively weaker adaptive capacity than during rest periods. Furthermore, rMSSD, which serves as an indicator of cardiac function stability, decreased during the rest phase, potentially reflecting a state of concern or unease (Seo and Kim 2015). This may be linked to participants' feelings of confinement in a limited space during the resting period. Additionally, LF, which reflects sympathetic nervous system activity, showed a decrease, indicating a weakening of sympathetic nervous activity. This suggests heightened activation of the sympathetic nervous system during work, implying a more tense and excited state. In contrast, the resting phase likely led to a decrease in such sympathetic activation, signaling reduced excitement.

From a practical perspective, both light intensity and CCT have the potential to directly or indirectly influence individuals' working and resting states, particularly in confined and narrow environments. The use of LED lights with adjustable intensity and CCT in such spaces can significantly meet the varying needs of individuals during different work or rest phases. This strategic application of lighting aims to enhance and tailor the working and living conditions, aligning them more closely with the dynamic requirements of the individuals in that environment.

## 5 Limitations and Future Directions

First, although this study focused on confined spaces, which are increasingly utilized in industrial contexts for daily work and short‐term rest, the duration of light exposure in this study was relatively short compared to an actual 8‐h work shift. This limitation may have constrained the observed effects, particularly on physiological responses such as HRV. Previous research revealed that the effects of CCT on alertness and cognitive performance varied throughout the day (Li, Fang, Qiu, et al. 2024). Future studies should investigate the prolonged effects of light intensity and correlated color temperature (CCT) on alertness and cognitive performance to better simulate real‐world scenarios. Additionally, the long‐term impact of sustained exposure to specific lighting settings on health, mood, and productivity remains unclear. Longitudinal research is necessary to explore these extended effects and their implications.

Second, this study employed two levels of light intensity (300 and 500 lx) and three levels of CCT (2800, 5000, and 6500 K). While these settings align with practical indoor lighting standards, they may not comprehensively represent the full range of lighting conditions encountered in diverse environments. Future research could expand the scope of lighting conditions to include low‐light or extremely bright settings, providing a more nuanced understanding of the effects of light intensity and CCT. Furthermore, previous studies have highlighted the positive effects of dynamic lighting interventions on circadian rhythm regulation and sleep quality (Ru et al. 2023; Wang et al. 2022). Investigating the impact of dynamic lighting systems, which mimic natural light variations throughout the day, could offer valuable insights into optimizing lighting design for well‐being and productivity in confined spaces used for daily work and short‐term rest.

Third, the tasks used to assess working memory and alertness in this study, such as the N‐back and psychomotor vigilance task (PVT), may not fully capture the complexity of real‐world cognitive demands. In practical workplace scenarios, individuals often engage in multitasking and complex decision‐making processes. Future studies should incorporate tasks of varying difficulty levels and higher ecological validity to better understand how lighting conditions influence cognitive performance in real‐life contexts.

## 6 Conclusion

This study investigates the impact of varied lighting conditions, encompassing both light intensity and CTT, on an individual's working memory and alertness within confined spaces. Based on the laboratory study involving 30 people, the following conclusions can be drawn.

1. In terms of alertness, it was observed that subjective alertness during the working state experienced a combined impact from both light intensity and CTT. Participants demonstrated peak alertness under lighting conditions of 300 lx and 5000 K. However, subjective alertness during the resting state was not influenced by various lighting conditions. In contrast, objective measures revealed that a light intensity of 300 lx led to superior alertness, with the 2800 K CTT outperforming the 5000 K condition.
2. The accuracy of working memory related tasks was influenced by CTT, with heightened precision under 5000 compared 6500 K. Regarding reaction time, task responses were swifter under a light intensity of 300 compared with the 500 lx condition. Importantly, no significant variations were noted in the collected physiological data under different lighting conditions.

## Ethics Statement

This study was carried out in accordance with the recommendations of the Human Research Ethics Committee of the Department of Psychology at Zhejiang University of Technology [2024D004]. The procedures used in this study adhere to the tenets of the Declaration of Helsinki.

## Conflicts of Interest

The authors declare no conflicts of interest.

## Supporting information


Data S1.
