# Supplementary material for: The Impact of Lighting Conditions on Users' Alertness and Working Memory in Confined Spaces
Source: Psych J. 2025 Jun 10;14(4):614–29. doi: 10.1002/pchj.70022 (PMC12318591; doi:10.1002/pchj.70022)
Supplement: Supplementary file 1 — Data S1. [file PCHJ-14-614-s001.docx]

# Supplementary Materials

## Latin square experimental conditions

To provide a clearer understanding for readers, we have included a supplementary table illustrating the arrangement of experimental conditions for all participants. This table ensures transparency and helps clarify how the order effects were controlled in the study.

***Table S1. A 6 × 6 Latin-square design assigning six experimental*** ***conditions to participants.***

| Sequence | Order of experimental conditions | | | | | |
| --- | --- | --- | --- | --- | --- | --- |
| 1 | Condition1 | Condition2 | Condition6 | Condition3 | Condition5 | Condition4 |
| 2 | Condition2 | Condition3 | Condition1 | Condition4 | Condition6 | Condition5 |
| 3 | Condition3 | Condition4 | Condition2 | Condition5 | Condition1 | Condition6 |
| 4 | Condition4 | Condition5 | Condition3 | Condition6 | Condition2 | Condition1 |
| 5 | Condition5 | Condition6 | Condition4 | Condition1 | Condition3 | Condition2 |
| 6 | Condition6 | Condition1 | Condition5 | Condition2 | Condition4 | Condition3 |

## HRV indicators calculation formula

***Table S2. HRV indicators calculation formula.***

| **Parameters** | **Calculation** | **Remark** |
| --- | --- | --- |
| HR | 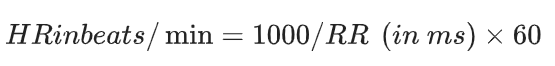 | RR interval is typically measured in milliseconds (ms). |
| SDNN | 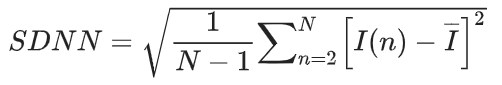 | I(n) represents the n-th RR interval (the time between consecutive heartbeats). |
| RMSSD | 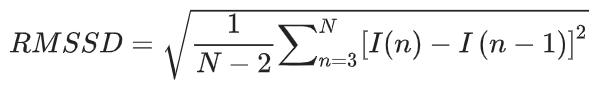 | I(n) and I(n−1) represent the n-th and (n−1) -th RR intervals. |
| NN50 | 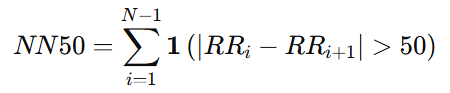 | RRi and RRi+1 represent the i-th and (i+1) -th RR intervals. |
| HF | embraces the PSD between 0.15 Hz and 0.4 Hz. |  |
| LF | represents the power of the periodic oscillations of the heart rate signal between the 0.04 Hz and 0.15 Hz frequencies. |  |
| LF/HF | reflects the global sympathicovagal balance |  |

**References:**

Albinet, C. T., Boucard, G., Bouquet, C. A., & Audiffren, M. (2010). Increased heart rate variability and executive performance after aerobic training in the elderly. *European journal of applied physiology*, *109*, 617-624.

Muñoz, J. E., Pereira, F., & Karapanos, E. (2016, September). Workload management through glanceable feedback: The role of heart rate variability. In 2016 IEEE 18th International Conference on e-Health Networking, *Applications and Services (Healthcom)* (pp. 1-6). IEEE.
